# Gas Evolution Kinetics in Overlithiated Positive Electrodes and its Impact on Electrode Design

**DOI:** 10.1002/advs.202400568

**Published:** 2024-04-06

**Authors:** Munsoo Song, Danwon Lee, Juwon Kim, Subin Choi, Ikcheon Na, Sungjae Seo, Sugeun Jo, Chiho Jo, Jongwoo Lim

**Affiliations:** ^1^ Department of Chemistry Seoul National University 1 Gwanak‐ro, Gwanak‐gu Seoul 08826 Republic of Korea; ^2^ Pohang Accelerator Laboratory 80 Jigok‐ro, Nam‐gu Pohang 37673 Republic of Korea; ^3^ LG Energy Solution R&D Center 188 Munji‐ro, Yuseong‐gu Daejeon 34122 Republic of Korea; ^4^ Institute of Applied Physics Seoul National University 1 Gwanak‐ro, Gwanak‐gu Seoul 08826 Republic of Korea

**Keywords:** electrode systems, gas evolution, kinetics, overlithiated positive electrodes, singlet oxygen

## Abstract

Increasing lithium contents within the lattice of positive electrode materials is projected in pursuit of high‐energy‐density batteries. However, it intensifies the release of lattice oxygen and subsequent gas evolution during operations. This poses significant challenges for managing internal pressure of batteries, particularly in terms of the management of gas evolution in composite electrodes—an area that remains largely unexplored. Conventional assumptions postulate that the total gas evolution is estimated by multiplying the total particle count by the quantities of gas products from an individual particle. Contrarily, this investigation on overlithiated materials—a system known to release the lattice oxygen—demonstrates that loading densities and inter‐particle spacing in electrodes significantly govern gas evolution rates, leading to distinct extents of gas formation despite of an equivalent quantity of released lattice oxygen. Remarkably, this study discoveres that O_2_ and CO_2_ evolution rates are proportional to ^1^O_2_ concentration by the factor of second and first‐order, respectively. This indicates an exceptionally greater change in the evolution rate of O_2_ compared to CO_2_ depending on local ^1^O_2_ concentration. These insights pave new routes for more sophisticated approaches to manage gas evolution within high‐energy‐density batteries.

## Graphical abstract

This study explores the impact of electrode configuration on gas evolution in high‐energy‐density batteries, Inter‐particle spacing depending on electrode loading densities significantly impacts the extent of O_2_ and CO_2_ evolution. This phenomena is attributed to the proportional relationships between O_2_ and CO_2_ evolution rates and ^1^O_2_ concentration. This provides insights for managing gas evolution in composite electrodes for enhanced performance.

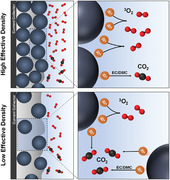

## 1 Introduction

As the rapid paradigm shift to electric vehicles (EVs) drives pressing demands for Li‐ion batteries as power sources, the development of positive electrode materials has progressed in pursuit of achieving high‐energy‐density.^[^ ^1^, ^2^, ^3^, ^4^, ^5^ ^]^ One of the few promising routes concerns increasing the lithium content within the lattice structure of positive electrode active materials, commonly referred to as overlithiated materials.^[^ ^6^, ^7^, ^8^ ^]^ However, this intensifies the irreversible release of oxygen atoms from the lattice structure at high degrees of delithiation,^[^ ^9^, ^10^, ^11^ ^]^ which has been recurrently held responsible for gas evolution reactions.^[^ ^12^, ^13^, ^14^ ^]^ This presents considerable challenges for the management of the internal pressure of batteries during operations due to the substantial accumulation of gas products in electrode systems over cycles. Consequently, this exacerbates safety concerns in batteries, such as swelling or rupture of cell casing.

In the complex electrochemical environment of a battery's positive electrode,^[^ ^15^ ^]^ the lattice oxygen is released as a reactive singlet oxygen (^1^Δ_g_ or ^1^O_2_). This reactive singlet oxygen can either evolve into inert triplet oxygen (^3^O_2_) through a self‐quenching process or form CO_2_ that initiates further electrolyte decomposition reactions.^[^ ^13^, ^14^, ^16^, ^17^ ^]^ The quantity of ^1^O_2_ released from each particle is critically determined by the intrinsic properties of the material during cycling.^[^ ^18^, ^19^ ^]^ However, the local consumption rate of ^1^O_2_ in each reaction pathway within composite electrodes, particularly in those producing O_2_ and CO_2_, holds even greater significance. This rate is crucial in determining the extent of gas evolution reactions and the consequent formation of side‐products, which, in turn, drive a variety of decomposition reactions.^[^ ^13^ ^]^ Despite its paramount importance, the fundamental principle governing the reaction kinetics of gas evolution in composite electrodes characterized by complex and intertwined particle networks remains largely underexplored. This gap in knowledge necessitates a comprehensive understanding in consideration of two major aspects: the response of each gas evolution rates to changes in local ^1^O_2_ concentration, and the influence of composite electrode configuration (i.e. particle‐particle distribution) on the local ^1^O_2_ environment and the overall gas evolution rates.^[^ ^14^ ^]^

To precisely establish the relationship between gas evolution rates and ^1^O_2_ concentration in composite electrodes, it is essential to methodically vary ^1^O_2_ concentration at the electrode level. This can be achieved by using an overlithiated catthode designed to release a tunable amount of ^1^O_2_ across various cycling conditions. Moreover, a selective assessment must be conducted to isolate the ^1^O_2_– driven gas evolution reactions from other potential gas–generating mechanisms that may concurrently occur at high potentials.^[^ ^20^, ^21^, ^22^, ^23^, ^24^, ^25^ ^]^ Consequently, the kinetics of gas evolution should be scrutinized in a scenario where ^1^O_2_ is abundantly released, thereby minimizing the interference from other types of gas evolution processes.

In this study, we utilize Li_6_CoO_4_—a compound known for the substantial release of lattice oxygen at low potential due to its labile oxygen states^[^ ^26^ ^]^—as a model system to probe the kinetics of gas evolution as a function of ^1^O_2_ concentration, within a voltage range from ≈3.5 to 4.3 V vs Li/Li^+^ that is compatible for conventional battery electrolytes. To discern the correlation between gas evolution rates and ^1^O_2_ concentration, we quantitatively analyzed the evolution of O_2_ and CO_2_ at various ^1^O_2_ levels using online electrochemical mass spectrometry (OEMS). We observed that O_2_ evolution, resulting from the self‐quenching of ^1^O_2_, showed a quadratic relationship with increasing ^1^O_2_ concentration, indicative of a second‐order reaction. In contrast, CO_2_ evolution, arising from the interaction of the electrolyte with ^1^O_2_, appeared to conform a first‐order reaction relative to ^1^O_2_ concentration. Remarkably, our results revealed that the distribution of products from competing reactions can be influenced by the local ^1^O_2_ concentration, which is affected by the distribution of ^1^O_2_–releasing particles within a composite electrode, despite the equivalence in average amount. Therefore, we conclude that the extent of interrelated gas evolution reaction is intricately linked to both the ^1^O_2_ concentration and the configuration of composite electrodes.

## 2 Results and Discussion

### 2.1 Release of Lattice Oxygen in Li_6_CoO_4_ During the Initial Charge

**Figure 1:**
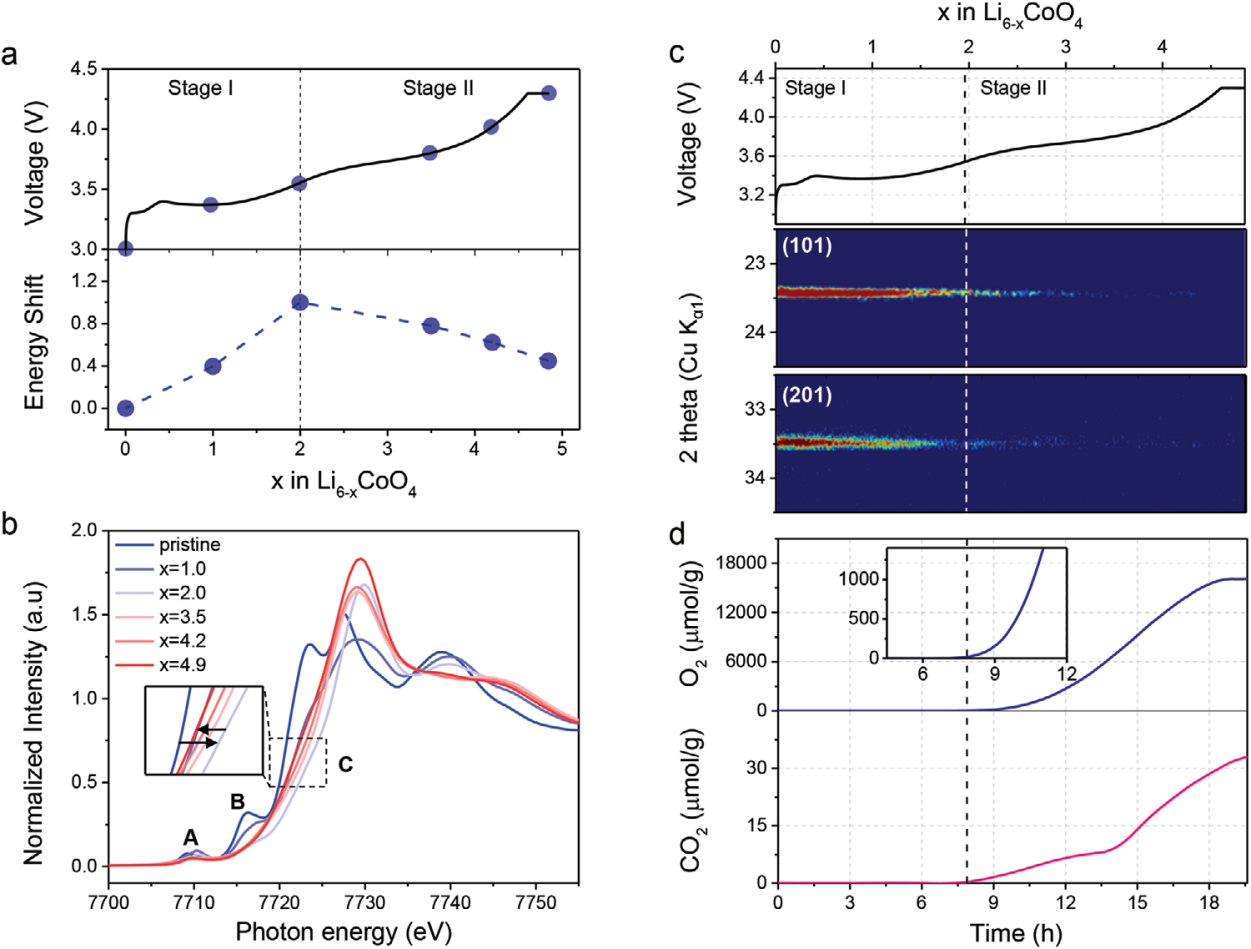
a) Galvanostatic profile of Li_6_CoO_4_ during charge to 4.3V vs. Li/Li^+^ and changes in half‐height position at different state of charge obtained from X‐ray absorption near edge spectroscopy of Co K‐edge. b) X‐ray absorption near edge spectroscopy (XANES) of Co K‐edge at different state of charge. c) in situ XRD contour maps of (101) and (201) diffraction peaks that indicates anti‐fluorite structure. d) O_2_ and CO_2_ evolution measured by OEMS quantitative in situ analysis.

Li_6_CoO_4_, classified as an overlithiated material, serves as an effective sacrificial positive electrode that compensates the lithium loss at counter electrodes during the initial cycle through its capability of releasing a substantial amount of lithium.^[^ ^27^, ^28^, ^29^ ^]^ Li_6_CoO_4_ locally has a distorted LiO_4_ tetrahedron in its anti‐fluorite structure that leads to a large density of labile oxygen states below the Fermi level, and hence it exploits improved electrochemical activity of lattice oxygen.^[^ ^26^, ^30^ ^]^ The galvanostatic profile of Li_6_CoO_4_ during the initial charge to 4.3 V versus Li/Li^+^ showcases two distinct plateaus at ≈3.3 and 3.6 V vs. Li/Li^+^, as presented in **Figure** **1**a. The first plateau is ascribed to the extraction of Li‐ions concurrent with the oxidation of Co, which is confirmed by X‐ray absorption spectroscopy (XAS) and X‐ray absorption near edge spectroscopy (XANES) analyses that characterize the oxidation state of Co (Figure 1a,b). However, the subsequent plateau exhibits a reduction of Co, suggesting an O‐to‐Co charge transfer process that coincides with the release of lattice oxygen. This process likely contributes to the breakdown of Co−O coordination in Li_6_CoO_4_, as shown by diminishing intensities of peaks A and B that correspond to 1s→4p and 1s→3d transitions, respectively.^[^ ^31^, ^32^ ^]^ The release of lattice oxygen, which is inferred from these observations, can be deduced from its implication in gas evolution and structural degradation.

To confirm the release of lattice oxygen, we monitored the structural changes in Li_6_CoO_4_ alongside gas evolution during the initial charging. Powder X‐ray diffraction (XRD) revealed that Li_6_CoO_4_ has an anti‐fluorite structure with a tetragonal P42/nmc space group as shown in Figure S1 (Supporting Information).^[^ ^26^, ^27^ ^]^ In situ XRD analysis captured the progressive fading of the main diffraction peaks, which corresponds to (101) and (201) planes. These diffraction peaks gradually faded toward the end of the first plateau and completely vanished by the end of the second plateau as shown in Figure 1c. This loss of long‐range ordering in the crystal structure aligns with the onset of O_2_ and CO_2_ (Figure 1d). Therefore, we deduce that the substantial gas evolution and associated degradation in the anti‐fluorite structure of Li_6_CoO_4_ is predominantly due to the release of lattice oxygen.

### 2.2 Release of Lattice Oxygen in Li_6_CoO_4_ during the Initial Charge

**Figure 2:**
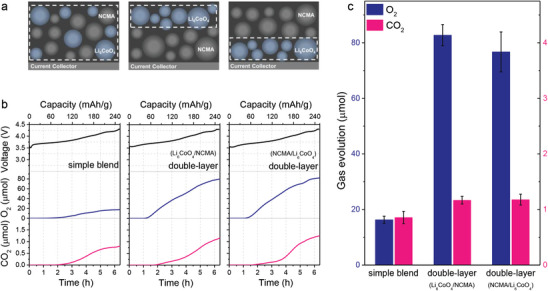
Change in extents of gas evolution depending on the configuration of electrode. a) The schematic of electrodes prepared in different configurations. b) Voltage profiles and corresponding O_2_ and CO_2_ evolution curves of the homogeneously distributed simple‐blend composite electrode and the bilayer composite electrodes of Li_6_CoO_4_ and NCMA. c) The change in amount of O_2_ and CO_2_ evolution depending on the configuration of electrode.

Building on our findings that the release of lattice oxygen results at 3.6 V vs. Li/Li^+^, the quantity of lattice oxygen released per mole of Li_6_CoO_4_ should theoretically remain constant under identical operational conditions. However, we found that the degree of gas evolution, which hinges on the reaction rates, appears to be substantially influenced by the concentration of reactants varying across different electrode configurations. To explore this further, we constructed electrodes with Li_6_CoO_4_ and NCMA (LiNi_x_Co_y_Mn_z_A1_1‐x‐y‐z_O_2_, x >0.85) in two separate configurations: a double‐layer composite with densely packed Li_6_CoO_4_, and a blend composite where Li_6_CoO_4_ particles are dispersed throughout the electrode volume (**Figure** **2**a; Figures S2 and S3, Supporting Information). The areal loading of the electrode composite was consistently maintained at 1.2 mg cm^−2^ for all electrodes. The active materials, constituting 97.5% of the electrode composite by weight, consisted of 3% Li_6_CoO_4_ and 97% NCMA. The density of Li_6_CoO_4_ was 0.077 g cm^−3^ for the simple blend electrode whereas it was 0.360 g cm^−3^ for the double‐layer electrode, as estimated from the thickness obtained from cross‐sectioned SEM images (Figures S2 and S3, Supporting Information).

The comparative analysis of O_2_ and CO_2_ evolution in these configurations during the initial charging (Figure 2b,c) showed that, despite an equal amount of Li_6_CoO_4_, the double‐layer composite electrode exhibits significantly greater evolution of both O_2_ and CO_2_. This phenomenon persisted regardless of the Li_6_CoO_4_ layer's position within the double‐layer structure, suggesting that the effect density of Li_6_CoO_4_ – a reflection of particle aggregation – plays a critical role in gas evolution dynamics. With NCMA showing minimal gas evolution (Figure S4, Supporting Information),^[^ ^14^, ^33^ ^]^ its dilution effect of ^1^O_2_ from Li_6_CoO_4_ is apparent. This allows to deduce that electrodes with varying effective densities and distribution of Li_6_CoO_4_ provide distinct environments that influence the local ^1^O_2_ concentration, which consequently leads to changes in the rates of competing reaction pathways. Therefore, it is imperative to establish the kinetics of gas evolution reactions and comprehend their implications on the electrode design.

**Figure 3:**
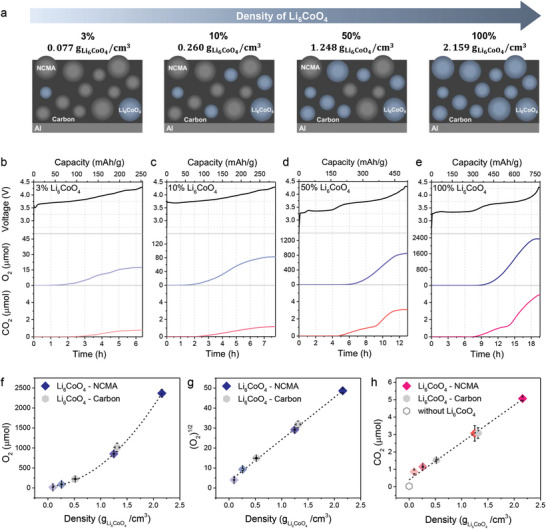
a) The schematic of electrodes in varying densities of Li_6_CoO_4_. Voltage profiles and corresponding O_2_ and CO_2_ evolution curves of the electrode with b‐e) 3, 10, 50, and 100 weight % of Li_6_CoO_4_. f) The amount of O_2_ evolution as a function of the density of Li_6_CoO_4_ with a fitted polynomial curve that indicates a quadratic relationship. g) Square root of cumulative O_2_ evolution as a function of the density of Li_6_CoO_4_ with a linearly fitted line. h) Cumulative CO_2_ evolution as a function of the density of Li_6_CoO_4_ with a linearly fitted line. CO_2_ evolution measured in the electrode solely composed of NCMA (open hexagon) was included in linearly fitted line to consider CO_2_ evolved under absence of Li_6_CoO_4_.

To establish a more concrete correlation between the gas evolution and ^1^O_2_ concentration, we quantitatively assessed the behavior of O_2_ and CO_2_ evolution across varying ^1^O_2_ concentrations. We could systematically modulate the ^1^O_2_ concentration by adjusting the Li_6_CoO_4_ density within electrodes, while maintaining areal loading and electrode thickness constant (**Figure** **3**a). Scanning electron microscopy (SEM) images confirmed the homogeneous distribution of Li_6_CoO_4_ (Figure S5, Supporting Information). All electrodes were charged at a consistent current density of 40 mAg^−1^ to maintain uniform electrochemical conditions for Li_6_CoO_4_ particles within composite electrodes. Our test across all electrodes showed consistent onset potential for gas evolution (O_2_ and CO_2_) and complete capacity utilization for both NCMA and Li_6_CoO_4_ (Figure 3b–e), indicating a consistent release of ^1^O_2_ per mole of Li_6_CoO_4_.

The relationship between O_2_ evolution and Li_6_CoO_4_ density during the initial charge is plotted in Figure 3f. A quadratic increase in O_2_ evolution was observed with rising Li_6_CoO_4_ density, fitting a second‐degree polynomial trend. This trend is further substantiated by a linear correlation between the square root of detected O_2_ and Li_6_CoO_4_ density (Figure 3g). Even with modifications to density by adding conductive carbon instead of NCMA (Figure S6, Supporting Information), the quadratic relationship still persisted. This suggests that the O_2_ evolution rate escalates with the Li_6_CoO_4_ density while maintaining a consistent rate constant. Therefore, O_2_ evolution, driven by the self‐quenching of ^1^O_2_, adheres to a second‐order reaction in relation to the reactant concentration.

Figure 3h illustrates the CO_2_ evolution during the initial charge, plotted as a function of the density of Li_6_CoO_4_. In contrast to O_2_ evolution, CO_2_ evolution exhibits a directly proportional increase with Li_6_CoO_4_ density. The NCMA electrode without Li_6_CoO_4_ only produces 0.027 µmol of the total CO_2_ evolution (Figure S4, Supporting Information), representing a negligible amount in comparison to CO_2_ from Li_6_CoO_4_. This suggests that the predominant source of CO_2_ is the chemical oxidation of electrolyte solvent by the release of ^1^O_2_, aside from minimal CO_2_ generation from other sources such as electrochemical oxidation of electrolyte or the decomposition reaction of surface impurities.^[^ ^25^, ^34^, ^35^, ^36^ ^]^ Consequently, CO_2_ evolution mediated by the reaction with ^1^O_2_ adheres to a first‐order reaction. This linear increase also suggests that the initial interaction between ^1^O_2_ and electrolyte solvent may serve as the rate‐determining step within the CO_2_ evolution pathway, which involves several intermediate steps.

Throughout the course of CO_2_ evolution pathway, a variety of side‐products are invariably generated and incorporated in the electrolyte, involving a series of intermediate steps.^[^ ^13^, ^16^ ^]^ The identification of these side‐products in the liquid phase is critical for delineating the prevailing reaction pathway for CO_2_ evolution and for mitigating the confounding effects of concurrent side reactions on the measurement of gas evolution rates. Hence, we employed ^1^H nuclear magnetic resonance (NMR) spectroscopy to detect and analyze these side‐products in the liquid phase, thereby isolating and characterizing reaction rates of O_2_ and CO_2_ evolution without an interference from ancillary reactions.

Notable side‐products produced in the presence of Li_6_CoO_4_ were glycolic acid (3.98 ppm) and formic acid (8.16 ppm) as shown in Figure S7 (Supporting Information). Glycolic acid is presumably formed through the decomposition of ethylene carbonate (EC), where ^1^O_2_ deprotonates the carbon atom in the EC ring to produce water and an aldehyde‐containing intermediate. This intermediate is then hydrolyzed, yielding glycolic acid and CO_2_.^[^ ^13^, ^16^ ^]^ Similarly, the formation of formic acid is initiated by ^1^O_2_ deprotonating the methyl group on dimethyl carbonate (DMC), leading to the formation of an intermediate that, upon hydrolysis, produces formic acid and CO_2_ as the end products.

**Figure 4:**
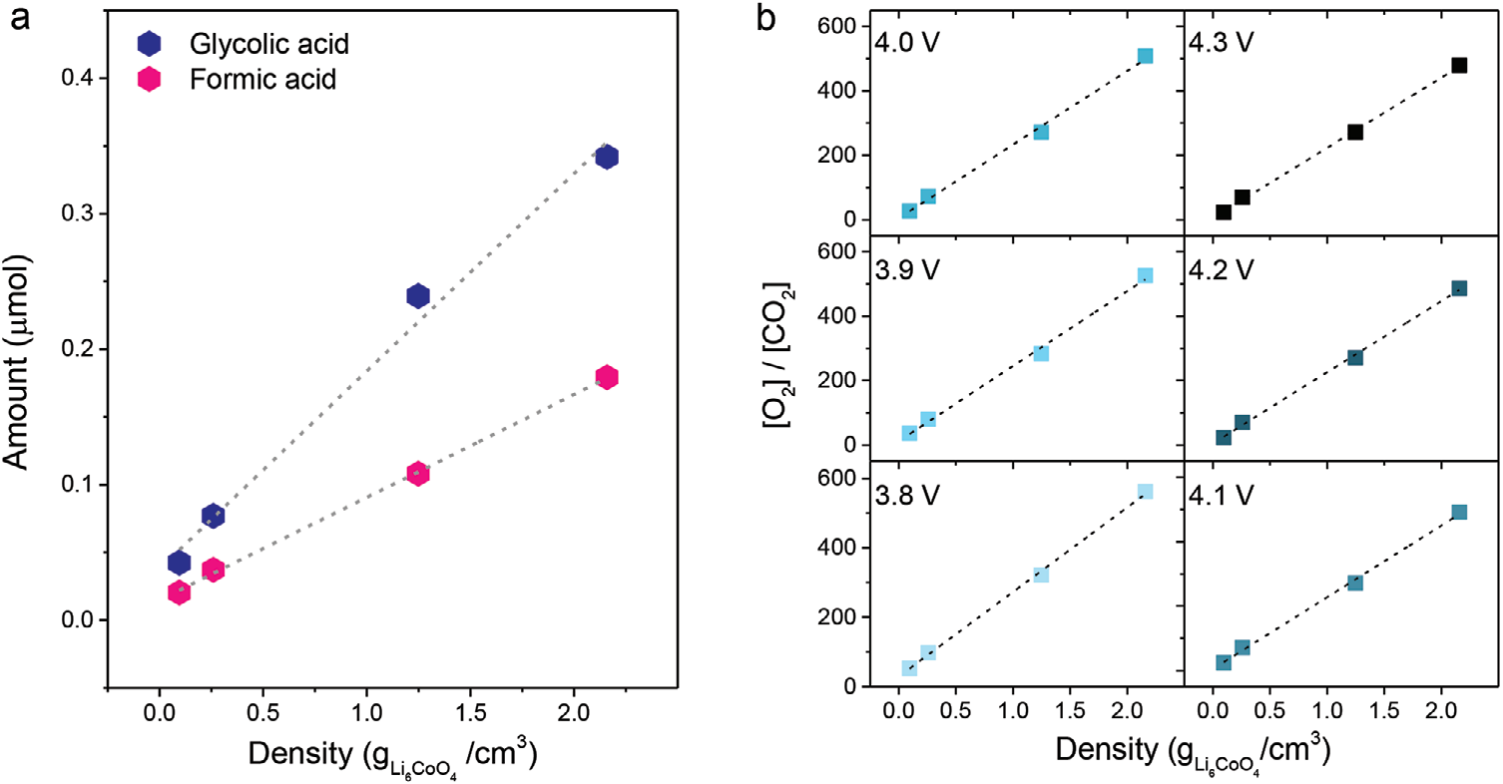
a) Quantified amounts of glycolic acid and formic acid that readily form as side products of reactions between ^1^O_2_ and electrolyte solvents. The amount of glycolic acid and formic acid is plotted as a function of the Li_6_CoO_4_ density. b) the ratio of evolved O_2_ and CO_2_ with respect to the density of Li_6_CoO_4_ at six different points within the potential range from 3.8 to 4.3 V versus Li/Li^+^ during the initial charge.

We quantified the amount of glycolic acid and formic acid by measuring their integrated peak area against an internal standard in ^1^H NMR spectra, which correlates with the number protons in the sample volume.^[^ ^37^ ^]^ The amounts of glycolic acid and formic acid rose linearly with increasing density of Li_6_CoO_4_ (**Figure** **4**a), establishing the direct relationships with ^1^O_2_ concentration. These findings substantiate that the observed liquid products and CO_2_ are products of the same reaction pathway, which follows a first‐order reaction pattern with respect to ^1^O_2_. This further suggests that the initial interaction between ^1^O_2_ and electrolyte solvent molecules likely constitute the rate‐determining step in the reaction pathway of CO_2_ evolution.

### 2.3 Relative Amount of O_2_ and CO_2_ Depending on the Local ^1^O_2_ Concentration, Controlled by the Particle‐Particle Distance and Distribution within an Electrode

The distribution of products from competing gas evolution reactions is governed by the proportion of ^1^O_2_ consumed in each reaction, which reflects their reaction rates. Notably, O_2_ evolution follows a second‐order reaction, as physical quenching of ^1^O_2_ into ^3^O_2_ requires two ^1^O_2_ molecules in (Equation 1).^[^ ^18^ ^]^ On the other hand, CO_2_ evolution follows a first‐order reaction (Equation 2). By examining the ratio of evolved O_2_ and CO_2_ relative to the Li_6_CoO_4_ density, a direct correlation between the rates of O_2_ and CO_2_ evolution and ^1^O_2_ concentration [^1^O_2_] can be deduced (Equation 3). The symbol “r” is the evolution rate, “k” is the rate constant, and “n” is the evolved amount.

(1)

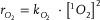



(2)

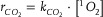



(3)

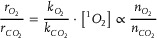




Figure 4b presents the linear correlation between the [O_2_]/[CO_2_] ratio and Li_6_CoO_4_ density during the initial charge, spanning the entire potential range from 3.8 to 4.3 V vs. Li/Li^+^, with a 4.2% deviation in their slopes. This persistence of distinct reaction orders for O_2_ and CO_2_ across various electrochemical biases confirms that the evolution rates of O_2_ and CO_2_ are governed by chemical kinetics rather than electrochemical processes, as long as ^1^O_2_ is released and participates in the chemical reactions. Additionally, these findings suggest that a greater fraction of ^1^O_2_ is utilized in O_2_ evolution as opposed to CO_2_ evolution, particularly when ^1^O_2_ concentration is locally high within the matrix of composite electrodes.

**Scheme 1:**
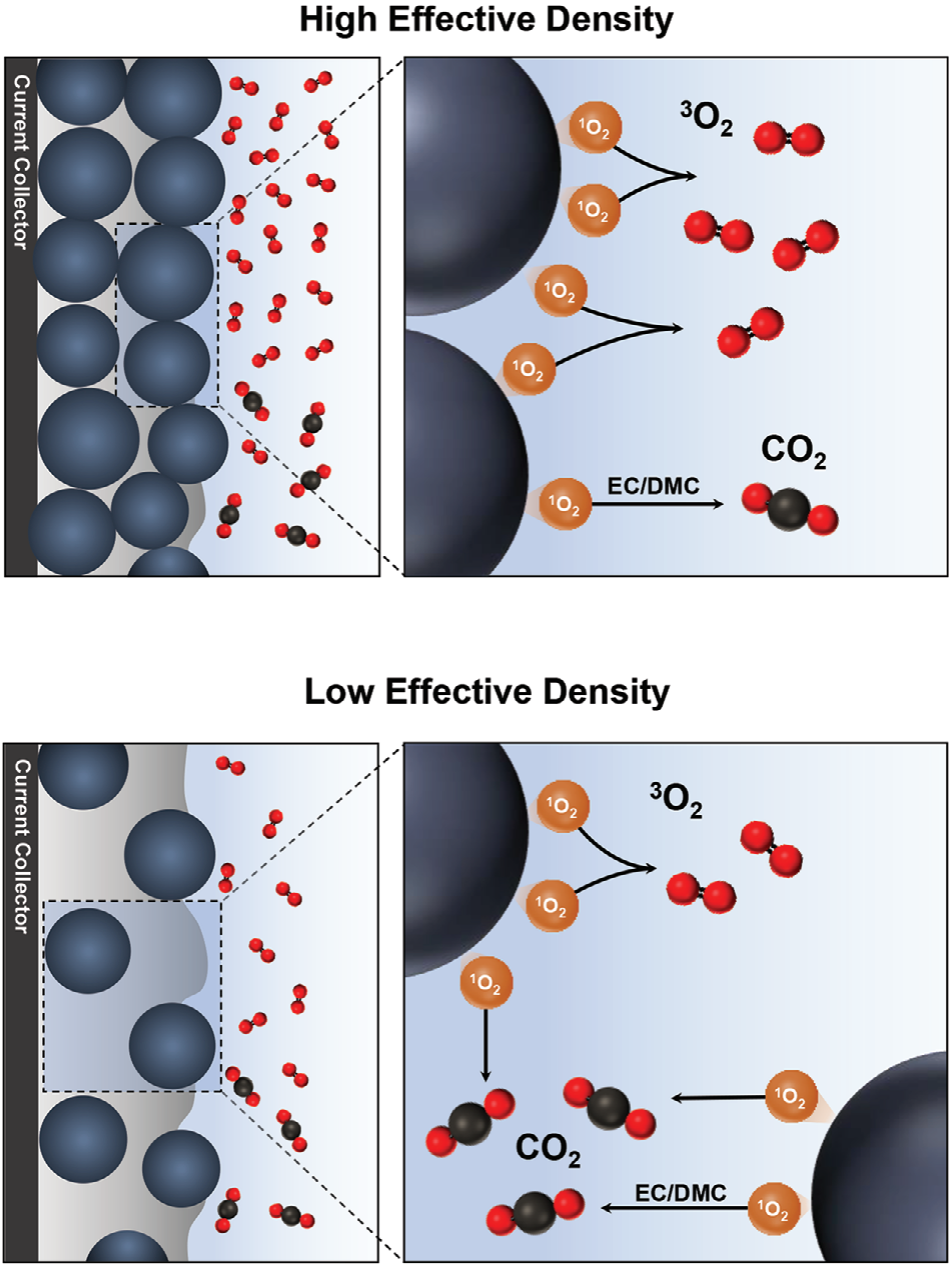
The contrasting product distributions based on the local concentration of ^1^O_2_ linked to electrode configuration, particularly in conjunction with the loading density and inter‐particle spacing within an electrode.

Overall, the extents of O_2_ and CO_2_ evolution may significantly vary depending on the electrode configuration (**Scheme** **1**). While the amount of ^1^O_2_ released from an individual particle likely remains constant, the local environment pertaining to ^1^O_2_ is largely dependent on the loading density and inter‐particle spacing within electrodes. As the evolution rates of O_2_ and CO_2_ are proportional to the concentration of ^1^O_2_ in quadratic and linear dependencies, respectively, the extents of O_2_ and CO_2_ evolution evolve contrastingly in response to changes in the local concentration of ^1^O_2_. This, in turn, results in the distinctive product distribution depending on the electrode configuration. Thus, in the design of electrodes, it becomes imperative to employ more sophisticated approaches that consider not only the intrinsic properties of materials but also gas evolution kinetics based on local environments in conjunction with electrode configurations.

## 3 Conclusion

We comprehensively investigated the gas evolution kinetics in Li_6_CoO_4_—a system known to facilely release lattice oxygen—and their implications for electrode configurations. This study found that gas evolution rates significantly shaped by the design of the electrode, especially by the loading density and spatial arrangement of particles within it. By systematically establishing the relationship between the extents of gas evolution and the concentration of ^1^O_2_, we discovered that the rates of O_2_ and CO_2_ evolution are correlated with the concentration of ^1^O_2_ in second and first order, respectively. It became evident that the extent of O_2_ and CO_2_ evolution may vary with the inter‐particle spacing, even when the number of particles remains constant within the electrode. The different evolution rates in response to the local ^1^O_2_ concentration underscore the impact of particle distribution on gas generation. This comprehensive examination of gas evolution kinetics in the context of overlithiated positive electrode configurations offers valuable insights for the strategic design of electrodes. Such designs aim to control the product distribution from competing reactions, optimizing the performance based on our understanding of reaction kinetics.

## 4 Experimental Section

### Electrode Preparation and Electrochemical Operation

Pristine Li_6_CoO_4_, and LiNi_x_Co_y_Mn_z_A1_1‐x‐y‐z_O_2_ (x > 0.85) powders were provided by LG Energy Solution. The powder X‐ray Diffraction (XRD) pattern of pristine Li_6_CoO_4_ was measured via in‐house XRD instrument (D8 Advance, Bruker) in the 2*θ* range of 15°–60° with a step size of 0.02° and step time of 3 s. Particles and electrode images were collected by FESEM (Apreo 2 S Hivac, Thermofisher Scientific) with an accelerating voltage of 15 kV. For electrode distribution analysis using FESEM, electrode samples were prepared using the ion‐milling system (IM4000, Hitachi) with 6 kV accelerating voltage at 60° for 30 min at the Research Institute of Advanced Materials, Seoul National University.

Pristine Li_6_CoO_4_ powder, polyvinylidene fluoride (PVDF), and Super P (TIMCAL) in the ratio of 97.5:1:1.5 wt.% were homogeneously dispersed in *N*‐methyl‐pyrrolidone (Acros Organics, 99%) in a planetary mixer (Thinky) at 2000 rpm for 15 min. The positive electrode composite was casted on an aluminum current collector with the thickness of 80 µm using a doctor blade and left to dry for 12 h under dry air condition of 80 °C. The areal loading of composite was 1.2 mg cm^−2^ after completely drying, and electrodes were pressed to 45 µm by calendaring. To prepare electrodes in varying densities of overlithiated materials, Li_6_CoO_4_ was replaced with NCMA in different weight percentage while maintaining the areal loadings and thickness of electrode. All electrodes were transferred to an argon‐filled glovebox ([O2] <1 ppm, [H2O] <0.1 ppm) for storage and assembly of any types of electrochemical cells used in experiments. 2032‐type coin cells were assembled with 12 mm positive electrode, 17 mm separator, 14 mm lithium metal counter electrode, and 1M LiPF_6_ EC/DMC (1:1 volume ratio). For all electrochemical tests, a galvanostatic cycling was performed at a rate of 40 mAg^−1^ to 4.3 V vs. Li/Li^+^, followed by a constant voltage step until the current reaches 10 mAg^−1^.

### Operando Gas Analysis

Online differential mass spectrometry (HPR‐40, Hiden Analytical) was modified to accommodate gas analysis on Li‐ion batteries. OEMS experiments were performed with lithium metal counter electrodes and positive electrodes at varying densities of overlithiated materials, implemented in a custom‐built OEMS cell platform. The volume of 1M LiPF_6_ EC/DMC (1:1 volume ratio) electrolyte added to the cell was maintained at 1 mL. Electrochemical data were obtained with VSP‐200, and signals from mass spectrometer were collected with Hiden analytical software. All OEMS experiments proceeded after 1 hour open‐circuit‐voltage to stabilize background signals from the OEMS instrument. For quantitative analysis of gas evolution, signals obtained from the mass spectrometry were quantified using the 3D calibration surface contour that was constructed based on signals and partial pressures of standard gases in various concentrations.

### Operando X‐Ray Diffraction Anaylsis

Operando XRD experiments were performed using pouch cells. Pouch cells were ≈5cm by 8cm, and the components consisting of pouch cells were analogous to coin cell as specified. The pouch cells were assembled with a lithium metal counter electrode, glass fiber separator, and Li_6_CoO_4_ positive electrode with 1M LiPF_6_ EC/DMC (1:1 volume ratio) electrolyte. Operando XRD patterns were collected by an in‐house XRD instrument (D8 Advance, Bruker) in the 2*θ* range of 15°–60° with an average measurement interval of 2 min per images. Data reduction from 2D to 1D was performed with MATLAB, and the sample‐to‐detector distance was calibrated based on diffraction peaks from the aluminum current collector as the reference.

### Post‐Mortem Characterization

For X‐ray absorption spectroscopy experiments, electrodes were retrieved after disassembling coin cells at 5 different states of charge during the cycle with specified galvanostatic protocol. Retrieved electrodes were thoroughly washed with DMC to remove any electrolyte components that possibly hinder the accurate measurements, and electrodes were vacuum‐sealed in aluminum pouches. The X‐ray absorption near edge spectroscopy (XANES) at Co K‐edge was measured at the 7D XAFS beamline of Pohang Light Source (PLS). The energy shift was calibrated based on the Co metal foil as the reference. After fitting the linear background in the pre‐edge and post‐edge regions and subtracting from each spectrum, the spectra were normalized using the Athena software.

^1^H NMR spectroscopy (Ascend 500, Bruker) was incorporated for the analysis on byproducts formed in the electrolyte during the electrochemical operation under varying concentration of overlithiated materials. After the coin cells with electrodes in varying densities of Li_6_CoO_4_ were cycled with a specified galvanostatic cycling protocol, glass fibers separators were retrieved from disassembled coin cells. Each of the four electrolyte samples cycled under varying concentration of overlithiated materials as well as a pristine reference sample were prepared by extracting electrolyte soaked in glass fiber separators. Each ^1^H NMR spectra was acquired at 500 MHz frequency with 1024 scans, and dimethyl sulfoxide was used as the internal standard solvent for quantification of byproducts in the liquid phase.

## Conflict of Interest

The authors declare no conflict of interest.

## Supporting information

Supporting Information

## Data Availability

The data that support the findings of this study are available in the supplementary material of this article.
