# Supplementary material for: Gas Evolution Kinetics in Overlithiated Positive Electrodes and its Impact on Electrode Design
Source: Adv Sci (Weinh). 2024 Apr 6;11(22):2400568. doi: 10.1002/advs.202400568 (PMC11165528; doi:10.1002/advs.202400568)
Supplement: Supplementary file 1 — Supporting Information [file ADVS-11-2400568-s001.docx]

Supporting Information

Gas Evolution Kinetics in Overlithiated Positive Electrodes and its Impact on Electrode Design

Munsoo Song, Danwon Lee, Juwon Kim, Subin Choi, Ikcheon Na, Sungjae Seo, Sugeun Jo, Chiho Jo, and Jongwoo Lim*

M. Song, D. Lee, J. Kim , S. Choi, I. Na, S. Seo, and Prof. J. Lim

Department of Chemistry, Seoul National University, Seoul 08826, Republic of Korea.
E-mail: jwlim@snu.ac.kr

S. Jo
Pohang Accelerator Laboratory, 80 Jigok-ro, Nam-gu, Pohang 37673, Republic of Korea.

C. Jo
LG Energy Solution R&D Center, 188 Munji-ro, Yuseong-gu, Daejeon 34122, Republic of Korea.

**Figure S1.** The morphology, chemical composition, and structure of Li_6_CoO_4_. a) Field Emission Scanning electron microscopy (FESEM) image of Li_6_CoO_4_ and corresponding energy dispersive X-ray spectroscopy (EDS) of Co and O. b) XRD diffraction patterns of pristine Li_6_CoO_4_ powder. c) A representative schematics of the anti-fluorite structure with a tetragonal P42/nmc space group.

Field emission electron scanning microscopy (FESEM) images and correlative energy dispersive X-rays spectroscopy (EDS) element mapping were collected to analyze morphology and chemical composition of Li_6_CoO_4_. Co and O are homogeneously distributed within the particles with the molar ratio of 26.5 % as estimated from EDS results XRD patterns of pristine Li_6_CoO_4_ powder validates the anti-fluorite structure belonging to a space group of tetragonal P42/nmc without the impurities. A representative schematic visualizes the anti-fluorite structure with a tetragonal P42/nmc space group that Li_6_CoO_4_ belongs.

**Figure S2.** a) cross-sectional SEM image of a double layer composite electrode consisted of Li_6_CoO in top layer and NCMA in bottom layer. b-c) and corresponding energy dispersive X-ray spectroscopy (EDS) of O and Ni, respectively.

**Figure S3.** a) cross-sectional SEM image of a double layer composite electrode consisted of NCMA in top layer and Li_6_CoO_4_ in bottom layer. b-c) and corresponding energy dispersive X-ray spectroscopy (EDS) of O and Ni, respectively.

**Figure S4.** Gas evolution induced by the electrochemical decomposition of electrolyte and NCMA. a) Galvanostatic profile during the initial charge to 4.3 V vs. Li/Li^+^ and corresponding O_2_ and CO_2_ evolution measured via OEMS *in-situ* analysis. b) The amount of O_2_ and CO_2_ accumulated during the initial charge.

**Figure S5.** Distributions of Li_6_CoO_4_ particles in the electrodes of varying density. a) A schematic of electrodes in varying densities of Li_6_CoO_4_. b-e) FESEM images of electrodes in varying densities of Li_6_CoO_4_ that confirm the homogeneous distribution of Li_6_CoO_4_ particles within the electrodes. f-p) corresponding energy dispersive X-ray spectroscopy (EDS) of Co, O, and Ni that verifies the homogenous distribution of Li_6_CoO_4_.

**Figure S6.** Galvanostatic profiles and corresponding O_2_ and CO_2_ evolution curves for a) electrode with 20 % Li_6_CoO_4_ and 78.5 % conductive carbon. b) electrode with 50 % Li_6_CoO_4_ and 48.5 % conductive carbon.

**Figure S7.** ^1^H nuclear magnetic resonance (^1^H NMR) spectrum of a) electrolyte extracted from the cell with varying densities of Li_6_CoO_4_ after the initial charge to 4.3 V vs. Li/Li^+^. b) pristine electrolyte sample and electrolyte extracted from the cell with NCMA electrode after the initial charge to 4.3 V vs. Li/Li^+^.
